# Inhibition of PKC activity blocks the increase of ET_B _receptor expression in cerebral arteries

**DOI:** 10.1186/1471-2210-6-13

**Published:** 2006-11-28

**Authors:** Marie Henriksson, Petter Vikman, Emelie Stenman, Saema Beg, Lars Edvinsson

**Affiliations:** 1 Division of Experimental Vascular Research, Clinical Sciences, Lund University, Lund, Sweden

## Abstract

**Background:**

Previous studies have shown that there is a time-dependent upregulation of contractile endothelin B (ET_B_) receptors in middle cerebral arteries (MCA) after organ culture. This upregulation is dependent on mitogen-activated protein kinases and possibly protein kinase C (PKC). The aim of this study was to examine the effect of PKC inhibitors with different profiles on the upregulation of contractile ET_B _receptors in rat MCA. Artery segments were incubated for 24 hours at 37°C. To investigate involvement of PKC, inhibitors were added to the medium before incubation. The contractile endothelin-mediated responses were measured and real-time PCR was used to detect endothelin receptor mRNA levels. Furthermore, immunohistochemistry was used to demonstrate the ET_B _receptor protein distribution in the MCA and Western blot to measure which of the PKC subtypes that were affected by the inhibitors.

**Results:**

The PKC inhibitors bisindolylmaleimide I, Ro-32-0432 and PKC inhibitor 20–28 attenuated the ET_B _receptor mediated contractions. Furthermore, Ro-32-0432 and bisindolylmaleimide I decreased ET_B _receptor mRNA levels while PKC inhibitor 20–28 reduced the amount of receptor protein on smooth muscle cells. PKC inhibitor 20–28 also decreased the protein levels of the five PKC subtypes studied (α, βI, γ, δ and ε).

**Conclusion:**

The results show that PKC inhibitors are able to decrease the ET_B _receptor contraction and expression in MCA smooth muscle cells following organ culture. The PKC inhibitor 20–28 affects the protein levels, while Ro-32-0432 and bisindolylmaleimide I affect the mRNA levels, suggesting differences in activity profile. Since ET_B _receptor upregulation is seen in cerebral ischemia, the results of the present study provide a way to interfere with the vascular involvement in cerebral ischemia.

## Background

The endothelins constitute a group of vasoactive peptides mainly produced by vascular endothelial cells [1]. There are two known endothelin receptors in mammals and both are found in the vascular wall. Normally, the endothelin A (ET_A_) receptors are situated on the smooth muscle cells in arteries where they give rise to contractions, while the ET_B _receptors are primarily seen on endothelial cells. An activation of the endothelial ET_B _receptors will lead to dilatation [2]. Interestingly enough, after organ culture of arteries [3], experimental ischemic stroke [4] and experimental subarachnoid hemorrhage [5] there is an upregulation of contractile ET_B _receptors located on the smooth muscle cells of cerebral arteries. We have revealed that this upregulation, when occurring in organ culture, is dependent on mitogen-activated protein kinases (MAPKs) [6] and possibly protein kinase C (PKC) [7,8].

PKC was first discovered in 1977 [9], and comprises a family of serine/threonine kinases, which is divided into the conventional, the novel and the atypical PKCs [10-12]. The PKCs participate in a wide variety of intracellular signalling cascades and are activated by different stimuli, such as growth factors, hormones and neurotransmitters [12].

The PKC inhibitors Ro-31-8220 and Ro-31-7549, used in previous studies of ET_B _receptor upregulation [7,8], have been shown to affect other intracellular signalling molecules, for example c-Jun N-terminal kinase and MAPK phosphatase-1 [13,14]. Consequently there is a risk that the effect of the PKC inhibitors may be due to inhibition of other pathways rather than the PKC signalling pathway.

The aim of this study was therefore to establish the involvement of PKC in the upregulation of contractile ET_B _receptors in rat middle cerebral arteries (MCAs) during 24 hours of organ culture. This duration has proven to be an optimal time for obtaining an upregulation of contractile ET_B _receptors [7]. Subsequently, we wanted to examine where in the process of upregulation the PKCs were involved. This was done using a variety of PKC inhibitors which affect PKC in different manners. The endothelin receptors were examined both functionally with myographs, and on a molecular level with real-time PCR. Immunohistochemistry was used to visualize the distribution of ET_B _receptors on the arterial smooth muscle cells. Finally, Western blot was used to examine the levels of different PKC subtypes in the arteries after incubation with the PKC inhibitors.

The general PKC inhibitors tested, all of which are cell permeable, had different effects on the vascular ET_B _receptor upregulation. Bisindolylmaleimide I (Bis I), Ro-32-0432 and PKC inhibitor 20–28 (PKCi 20–28) all significantly affected the sarafotoxin 6c (S6c, selective ET_B _receptor agonist) induced contractility. In addition, Ro-32-0432 decreased the ET_B _receptor mRNA levels, as did Bis I to some extent. PKCi 20–28 had no such effect. However, PKCi 20–28 did reduce the amount of ET_B _receptor protein in the arteries and also decreased the protein amount of the five PKC subtypes tested (α, βI, γ, δ and ε).

## Results

### Contractile experiments

**Figure 1:**
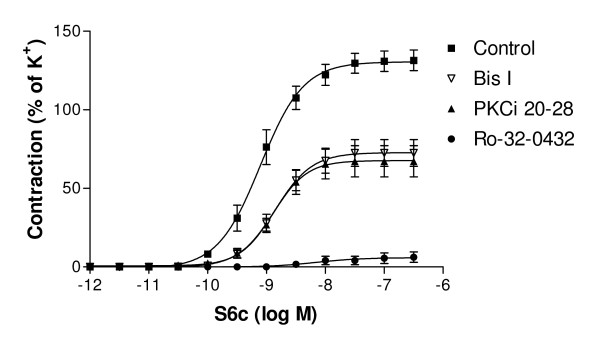
**Contractile response towards S6c**. Contractile responses towards the ET_B _receptor agonist S6c in MCAs incubated for 24 hours (control) and MCAs incubated for 24 hours with PKC inhibitors (Bis I, Ro-32-0432, PKCi 20–28). Each point represents mean value ± S.E.M. For statistical analysis, see Table 1.

K^+^-induced contractions did not differ between the control group and the arteries incubated with each of the PKC inhibitors (data not shown). Previous studies have shown that S6c does not induce contraction of fresh MCA segments [6,7]. After organ culture S6c produces contraction of cerebral arteries due to the enhanced expression of smooth muscle ET_B _receptor. In the present study, the S6c induced contraction was significantly decreased in the arteries cultured with the PKC inhibitors Bis I, Ro-32-0432 and PKCi 20–28 as compared to control (Table 1, fig. 1). Ro-32-0432 also affected the pEC_50 _value of the S6c dose response curve considerably (Table 1, P < 0.001). The PKC inhibitor chelerythrine chloride had no effect on the S6c induced contraction and was therefore not included in the subsequent experiments (Table 1). After addition of S6c, the ET_B _receptors are desensitized and a subsequent application of endothelin-1 (ET-1, ET_A_/ET_B _agonist) only affects the remaining contractile ET_A _receptors [15].

**Table 1:** Contractile responses to S6c and ET-1 in MCA incubated with four different PKC inhibitors

		**S6c**		**ET-1**	
	n	Emax (%)	pEC_50_	Emax (%)	pEC_50_
Control	10	131 ± 7	9.13 ± 0.10	152 ± 6	8.74 ± 0.12
Bisindolylmaleimide I	14	73 ± 8**	8.97 ± 0.08	143 ± 6	8.70 ± 0.08
Chelerythrine chloride	9	102 ± 7	8.95 ± 0.20	139 ± 6	8.77 ± 0.18
PKC inhibitor 20–28	14	67 ± 10**	8.80 ± 0.16	145 ± 7	8.64 ± 0.17
Ro-32-0432	9	6 ± 3***	8.92 ± 0.07***	194 ± 12	8.36 ± 0.09

*P < 0.05, **P < 0.01, ***P < 0.001 compared to control. *n *represents the number of artery segments.

In the present study we found that the PKC inhibitors did not have any effect on neither the maximum ET-1 induced contraction nor on the pEC_50 _value of the ET-1 concentration-response curve (Table 1, P > 0.05).

### mRNA analysis

mRNA was analyzed with real-time PCR and standard curves were produced for each primer pair showing that all samples were within the linear amplification range. The slopes of the curves were close to 3.3, thus the amplification was close to optimal (data not shown).

**Figure 2:**
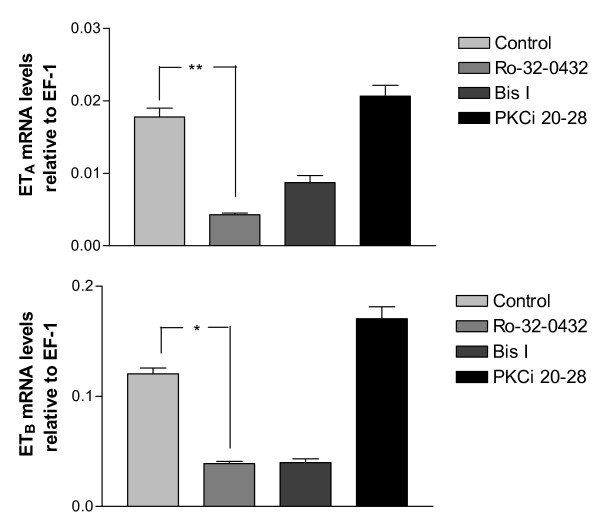
**Endothelin receptor mRNA levels**. ET_A _receptor and ET_B _receptor mRNA levels relative to EF-1. MCAs incubated for 24 hours (control) are compared to MCAs incubated for 24 hours with PKC inhibitors (Bis I, Ro-32-0432, PKCi 20–28). Data are presented as mean values ± S.E.M. *P < 0.05, **P < 0.01.

The results of the real-time PCR showed that Ro-32-0432 decreased the ET_A _receptor and ET_B _receptor mRNA levels significantly as compared to control (ET_A _receptor : 0.004 ± 0.0002 compared to 0.018 ± 0.001; ET_B _receptor: 0.039 ± 0.002 compared to 0.12 ± 0.005, Figs. 2A–B). Bis I behaved similarly in decreasing the ET_B _receptor mRNA levels, however not significantly (0.04 ± 0.003 compared to 0.12 ± 0.005, P = 0.0615, Fig 2B). PKCi 20–28 had no effect on the endothelin receptor mRNA levels (Figs. 2A–B).

### Receptor protein expression

**Figure 3:**
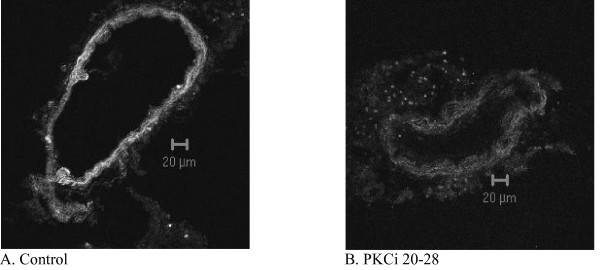
**Distribution of ET_B _receptor protein**. Expression of ET_B _receptor protein in a) MCA incubated for 24 hours (control) and b) MCA incubated for 24 hours with addition of PKCi 20–28.

Immunohistochemistry showed a clear decrease of ET_B _receptors in the arteries treated with PKCi 20–28 in comparison to control (57% ± 4% of the control value, P < 0.05, Fig. 3). There was a small decrease in ET_B _receptor density in the arteries treated with Ro-32-0432 (89% ± 9% of control value) and Bis I (75% ± 13% of control value), however it was not significant.

### PKC subtype expression

**Figure 4:**
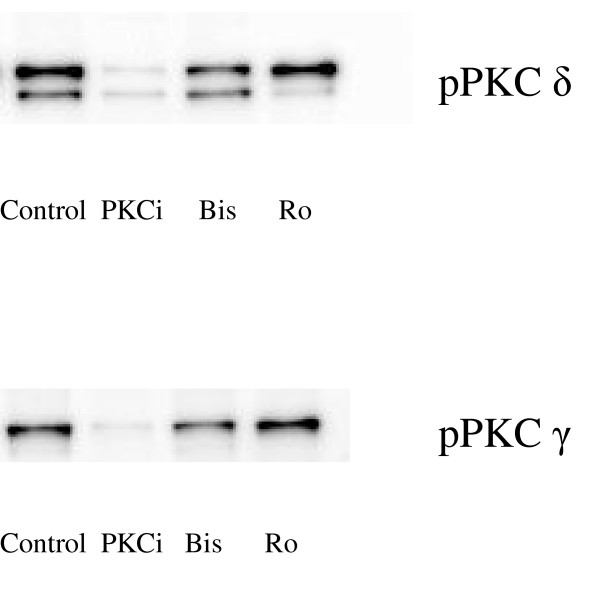
**PKC subtype expression**. Expression of phosphorylated PKCδ protein (A) and PKCγ protein (B) in MCAs incubated for 24 hours (control) and MCAs incubated for 24 hours with PKC inhibitors.

Western blot experiments were carried out using antibodies directed specifically against phosphorylated, and thereby activated, PKC isoforms (α, βI, γ, δ and ε). These tests showed that PKCi 20–28 was able to decrease the protein amount of all five PKC subtypes tested, although the decrease was most prominent for PKCδ (38% ± 20% of control, Fig. 4A) and PKCγ (51% ± 36% of control, Fig. 4B). Conversely Ro-32-0432 and Bis I did not decrease the protein levels of the PKC subtypes.

## Discussion

This study shows that each of the three general PKC inhibitors attenuates the organ culture induced upregulation of the ET_B _receptor mediated contraction seen in rat MCA. A fourth PKC inhibitor, chelerythrine chloride, had no effect on the contractile responses of the arteries, and consequently was not included in the additional experiments. A possible explanation to this lack of effect could be that chelerythrine chloride has been shown to alter vasoconstrictive responses through interaction with various phosphodiesterases and thus lacks specificity [16]. Furthermore, chelerythrine chloride activates c-Jun N-terminal kinase pathways [17]. Our group has recently discovered that c-Jun N-terminal kinase plays an important role in the upregulation of ET_B _receptors in porcine coronary arteries (Nilsson et al., unpublished data), which could explain why chelerythrine chloride fails to inhibit the ET_B _receptor upregulation in the present study.

Bis I and Ro-32-0432 are both competitive inhibitors for the ATP-binding site of PKC [18,19], but in a slightly different manner, which results in differences in PKC subtype affinity. Bis I inhibits the conventional PKCs (subtypes α, βI, βII and γ) with similar potency (IC_50 _= 10 nM) [18], and the subtypes δ and ε to a lesser extent [20]. Ro-32-0432 mainly affects the α subtype (IC_50 _= 9 nM), but also βI, βII and γ [19].

The third inhibitor tested, the PKC inhibitor 20–28, is a pseudosubstrate peptide, which mimics a particular domain of the PKC that keeps the enzyme in an inactive state [21]. The peptide is based on the pseudosubstrate motif of PKCα and β and is myristoylated to allow cell membrane permeability. All these three PKC inhibitors significantly reduced the organ culture induced upregulation of the ET_B _receptor mediated contraction.

The real-time PCR showed that Ro-32-0432 and, to a certain extent, Bis I blocked the elevated levels of ET_B _receptor mRNA relative to that seen in the control arteries. However, PKCi 20–28 failed to exhibit this effect on the mRNA level. Instead, PKCi 20–28 diminished ET_B _receptor protein expression. One reason for this discrepancy could be that Ro-32-0432 and Bis I exert their inhibitory effects earlier in the production of ET_B _receptors in the arteries, possibly upstream from gene level, causing a decrease of the ET_B _receptor mRNA. PKCi 20–28 on the other hand, affects the ET_B _receptor protein production and not the mRNA levels and hence at a translational level. In concert the PKCi 20–28 decreased the amount of ET_B _receptor protein as shown by immunohistochemistry, and marginally in the case of Bis I and Ro-32-0432. Nevertheless, a small decrease could be enough to diminish the functional response. Thus, the results could be interpreted to show a multiple role for PKC in the upregulation of ET_B _receptors. Another explanation for the difference between the mRNA levels and the protein levels in the case of PKCi 20–28 is that the inhibiting effect may not be as prolonged as for the two other inhibitors and subsequently, at the time point measured, the ET_B _receptor mRNA might be restored to its normal level.

The ET_A _receptor mRNA in rat cerebral arteries is not altered by 24 hours of organ culture [7]. Still, Ro-32-0432 did decrease the ET_A _receptor mRNA levels after incubation. This was not accompanied by a diminished ET_A _receptor mediated contraction. This discrepancy could be due to the fact that the mRNA levels do not necessarily reflect the presence of functional receptors, and at a later time point, the ET_A _receptor density will decrease.

Previous studies with the PKC inhibitors Ro-31-8220 and Ro-31-7549 indicate that the conventional PKC subtypes (α, β, γ) are involved in the ET_B _receptor alteration after organ culture [7,8]. The present study confirms this, but also shifts the focus slightly towards the PKCδ subtype. This is interesting, since several studies have shown PKCδ to be deleterious in experimental ischemia [22-24]. For example, treatment with PKCδ inhibitors decreases infarct size [25], and PKCδ knock-out mice show a decreased infarct size compared to wild type mice [26]. Further studies need to be done to define the role of PKCδ in our experimental setting.

We have previously shown that in both experimental focal ischemia and subarachnoid hemorrhage there is an ET_B _receptor upregulation in the MCA [4,5]. This upregulation may lead to enhanced contraction of the arteries, which causes an attenuation of blood and oxygen supply to the infarcted area, and subsequently an aggravation of the ischemic damage. Thus, according to our findings here, a beneficial effect of PKC inhibition in ischemia could in part be due to a reduction of the ET_B _receptor upregulation in the cerebral arteries.

## Conclusion

This study demonstrates that PKC inhibitors are able to decrease the ET_B _receptor contraction and expression in smooth muscle cells of MCA following 24 hours of organ culture. A similar upregulation of contractile ET_B _receptors has been seen in an experimental model of focal ischemia. The results of the present study can therefore be of significance in finding new therapeutic targets in cerebral ischemia.

## Methods

### Artery preparation and organ culture procedure

The experimental procedures were approved by the Ethics Committee for Laboratory Animal Experiments at Lund University (Application number M 217-03).

Male Wistar rats (350–400 g, M & B, Denmark) were anesthetized with CO_2 _and decapitated. The right and left MCA were removed and dissected free from surrounding tissue. The arteries were cultured for 24 hours in Dulbeccos modified Eagle's medium (DMEM), supplemented with penicillin (100 U/ml), streptomycin (100 μg/ml) and amphotericin B (25 μg/ml), at 37°C in humidified 5% CO_2 _in air. This induces a maximal ET_B _receptor upregulation [7]. The inhibitors were added to the medium before the incubation (Bis I, 10 μM; Ro-32-0432, 10 μM; chelerythrine chloride, 1 μM and PKCi 20–28, 100 μM). The choice of dose was based on K_i _values and on previous studies using the inhibitors. Pilot experiments were done to determine the optimal doses in this setting.

### Myograph experiments

After incubation, the arteries were cut into cylindrical segments. The segments were mounted on two 40 μm diameter stainless steel wires in a Mulvany-Halpern myograph (Danish Myo Technology A/S, Denmark) [27,28]. One of the wires was connected to a force transducer attached to an analogue-digital converter unit (ADInstruments, Hastings, UK). The other wire was attached to a movable displacement device allowing adjustments of vascular tension by varying the distance between the wires. The experiments were recorded on a computer by use of the software program Chart™ (ADInstruments, UK). The segments were immersed in a temperature-controlled (37°C) bicarbonate buffer (for composition, see below). The buffer was continuously gassed with 5% CO_2 _in O_2_, resulting in a pH of 7.4. The arteries were given an initial tension of 1.2 mN, and were allowed to adjust to this level of tension for 1 hour. The contractile capacity was determined by exposure to a potassium-rich (63.5 mM) buffer (for composition, see below). Concentration-response curves for the agonists S6c (ET_B _receptor agonist) and ET-1 (ET_A _and ET_B _receptor agonist) were obtained by cumulative application (10^-12^-10^-6.5 ^M). Following sarafotoxin 6c administration, the endothelin ET_B _receptors are desensitized, leaving only endothelin ET_A _receptors to interact with endothelin-1 [15].

#### Calculations

The E_max _values represent the maximum vascular contraction as response to S6c or ET-1 and were calculated as percentage of the contractile response towards 63.5 mM K^+^. The pEC_50 _values represent the negative logarithm of the concentration which elicits half-maximum response. Data are expressed as mean values ± S.E.M. Statistical analyses were performed with Kruskal-Wallis non-parametric test with Dunn's post-hoc test. P < 0.05 is considered significant. There were 3–5 rats in each group, with 1–4 segments from each. *n *refers to the number of arterial segments.

### Real-time PCR

Total cellular RNA was extracted from each MCA using the FastRNA Pro Green Kit following the suppliers' instructions. The resulting pellet was finally washed with 75% ethanol, air-dried and redissolved in diethylpyrocarbonate-treated water. Reverse transcription of total RNA to cDNA was carried out using the GeneAmp RNA PCR kit in a Perkin-Elmer DNA Thermal cycler, using random hexamers as primers. The reaction mixture was incubated at 25°C for 10 minutes, 42°C for 30 minutes, heated to 99°C for 5 minutes and chilled to 5°C for 5 minutes.

Real-time PCR was performed in a GeneAmp 5700 Sequence Detection System (Applied Biosystems, Foster City, CA, USA) using the GeneAmp SYBR^® ^Green kit with the cDNA synthesized above as template in a 50 μl reaction volume. A no template control was included in all experiments. This technique consists of an optic imaging system that is able to monitor the amount of DNA in each PCR cycle via the detection of a fluorescent dye binding to double-stranded DNA. The DNA levels of the genes of interest are compared to an endogenous standard. In this study, specific primers for the rat ET_A _and ET_B _receptors were designed as follows: ET_A _receptor, forward: 5'-ATTGCCCTCAGCGAACAC-3'; reverse: 5'-CAACCAAGCAGAAGACGGTC-3', ET_B _receptor forward: 5'GATACGACAACTTCCGCTCCA-3'; reverse: 5'-GTCCACGATGAGGACAATGAG-3'. Elongation factor-1 (EF-1) mRNA was used as an endogenous standard. The EF-1 primers were designed as follows: forward; 5'-GCAAGCCCATGTGTGTTGAA-3'; reverse: 5'-TGATGACACCCACAGCAACTG-3'. The real-time PCR was carried out as follows: 50°C for 2 minutes, 95°C for 10 minutes, 40 cycles of 95°C for 15 seconds and 60°C for 1 minute. Each sample was examined in duplicates.

#### Calculations

The amount of ET_A _receptor and ET_B _receptor mRNA was calculated as relative to the amount of EF-1 mRNA in the same sample by the formula: X_0_/R_0 _= 2^CtR-CtX^, where X_0 _= original amount of endothelin receptor mRNA, R_0 _= original amount of elongation factor-1 mRNA, CtR = C_T_-value for elongation factor-1 and CtX = C_T_-value for the endothelin receptor. Each sample was examined in duplicates and the mean values were used.

Data are expressed as mean values ± S.E.M. Statistical analyses were performed with Kruskal-Wallis non-parametric test with Dunn's post-hoc test. P < 0.05 is considered significant. *n *= 6–7, where *n *is the number of MCAs used.

### Immunohistochemistry

After incubation for 24 hours, the MCAs were placed onto Tissue TEK, frozen and subsequently sectioned into 10 μm slices. The primary antibodies used were rabbit-antihuman endothelin ET_B _receptor antibody, diluted 1:400, goat-antimouse endothelin ET_A _receptor antibody, diluted 1:100 and mouse-antirat CD31 antibody, diluted 1:200. The secondary antibodies used were donkey-antirabbit Cy™3 conjugated, diluted 1:100, donkey-antigoat Cy™2 conjugated, diluted 1:100, and donkey-antimouse Cy™5 conjugated, diluted 1:100. All dilutions were done in phosphate buffered saline with 10% fetal calf serum. The antibodies were then detected at the appropriate wavelength in a confocal microscope (Zeiss, USA). As control, only addition of secondary antibodies was used.

#### Calculations

The images were analysed using the ImageJ software [29]. The fluorescence in 4–6 different areas in each artery was measured and a mean value was calculated. These values are presented as percentage fluorescence in the treated groups compared to the control group, where the control group was set to 100%. Data are expressed as mean values ± S.E.M with three arteries from three different rats in each group. Statistical analyses were performed with Kruskal-Wallis non-parametric test with Dunn's post-hoc test. P < 0.05 is considered significant.

### Western Blot

After incubation, the MCAs were collected and placed on ice, homogenized in lysis-buffer with protease- and phosphatase inhibitors (for composition, see below). After 20 minutes incubation in lysis buffer on ice, homogenates were centrifuged at 4500 g for 10 min at 4°C and supernatant collected. Total protein concentration was determined using a BioRad DC kit (Hercules, CA, USA) and the absorbance was measured at 750 nm on a Genesys 10 spectrophotometer (Thermo, Waltham, MA, USA).

Lysates were dissolved in Tris-glycine SDS sample buffer (Invitrogen A/S, Taastrup, Denmark) and boiled for 5 minutes. Equal amounts of protein (10 μg/lane) were loaded on a 8% Tris-glycine gel (Invitrogen A/S, Taastrup, Denmark) and separated by SDS-polyacrylamide gel electrophoresis. Molecular weight markers (New England BioLabs, Ipswich, MA, USA) were loaded on each gel for protein band identification. After separation, proteins were transferred to a nitrocellulose membrane (BioRad, Hercules, CA, USA). Subsequently the membrane was blocked with 6.5% non-fat milk in Tween-TBS overnight at 4°C. Membranes were then incubated with the following primary antibodies: rabbit polyclonal anti-PKCα phosphospecific (1:1000 dilution), rabbit polyclonal anti-PKCδ phosphospecific (1:1000 dilution), rabbit polyclonal anti-PKCε phosphospecific (1:1000 dilution), rabbit polyclonal anti-PKCβI phosphospecific (1:1000 dilution), rabbit polyclonal anti-PKCγ phosphospecific (1:1000 dilution) or mouse polyclonal β-actin overnight at 4°C, followed by 3 × 5 minutes wash with Tween-TBS. Thereafter, the membranes were incubated with the appropriate secondary antibody: goat anti-rabbit IgG-horseradish peroxidase or goat anti-mouse IgG-horseradish peroxidase (1:5000) for 1 hour at room temperature, followed by 5 × 5 minutes wash with Tween-TBS. Levels of β-actin were used to confirm equal loading of the lanes. The membranes were developed using the Supersignal Dura kit (Pierce, Rockford, IL, USA) and visualized using a Fujifilm LAS-1000 Luminiscent Image Analyzer (Stamford, CT, USA).

#### Calculations

Protein lysates from the four different groups were compared. MCAs from three animals were pooled for each sample and each group consisted of three samples. Quantification of band density was performed with the electrophoresis computer analysis program Fujifilm Science Lab Image Gauge 4.0. The immunoblot optical density values were determined with repeated measurements and are presented as percentage activity in the groups treated with PKC inhibitors compared to the control group, where the control group was set to 100%. All values are relative to the respective β-actin band density. Data are expressed as mean values ± S.E.M.

### Drugs and chemicals

The bicarbonate buffer had the following composition (mM): NaCl 119; NaHCO_3 _15; KCl 4.6; MgCl_2 _1.2; NaH_2_PO_4 _1.2; CaCl_2 _1.5 and glucose 5.6, while the potassium-rich bicarbonate buffer composition was as follows (mM): NaCl 59.5; NaHCO_3 _15; KCl 63.5; MgCl_2 _1.2; NaH_2_PO_4 _1.2; CaCl_2 _1.5 and glucose 5.6.

The DMEM supplemented with penicillin (100 U/ml), streptomycin (100 μg/ml) and amphotericin B (25 μg/ml) was purchased from Gibco (Stockholm, Sweden).

Bis I, Ro-32-0432, chelerythrine chloride and PKCi 20–28 were purchased from Calbiochem (San Diego, CA, USA) and dissolved in dimethyl-sulfoxide. ET-1 and S6c (Alexis Biochemicals, Lausen, Switzerland) were dissolved in 0.9% saline with 0.1% bovine serum albumin.

FastRNA Pro Green Kit was purchased from BIO 101 (Carlsbad, CA, USA).

GeneAmp RNA PCR kit and GeneAmp SYBR^® ^Green kit were purchased from Applied Biosystems (Foster City, CA, USA).

For the immunohistochemistry, Tissue TEK was purchased from Gibco (Stockholm, Sweden). Rabbit-antihuman ET_B _receptor antibody was purchased from IBL (Gunma, Japan), goat-antimouse ET_A _receptor antibody from Santa Cruz Biotechnologies (Santa Cruz, CA, USA) and mouse-antirat CD31 antibody from Serotec (Oxford, UK). The secondary antibodies, donkey-antimouse Cy™5 conjugated, donkey-antirabbit Cy™3 conjugated and donkey-antigoat Cy™2 conjugated, were purchased from JacksonImmunoResearch (Gothenburg, Sweden).

The lysis-buffer for Western blot had the following composition: 10 mM Tris pH 7.4, 50 mM β-Glycerophosphate, 100 μM Na_3_VO_4_, 0.5% Deoxycholate, 1 mM EGTA, 1 mM EDTA, 1 mM NaF, 20 mM Na_4_P_2_O_7_, 1% Triton X-100, 1 mM DTT, 20 μM Pepstatin, 20 μM Leupeptin, 0.1 U/ml Aprotinin, 1 nM Calyculin and 1 mM PMSF.

The primary antibodies for the Western blot were rabbit polyclonal antibodies with following specificity: anti-PKCα phosphospecific (phosphorylated at threonine 638), anti-PKCδ phosphospecific (phosphorylated at serine 664), anti-PKCε phosphospecific (phosphorylated at serine 739), anti-PKCβI phosphospecific (phosphorylated at threonine 642) and anti-PKCγ phosphospecific (phosphorylated at threonine 655) were purchased from Biosource (Camarillo, CA, USA). The mouse polyclonal β-actin antibody was purchased from Sigma (Saint Louis, USA). The secondary antibodies; goat anti-rabbit IgG-horseradish peroxidase and goat anti-mouse IgG-horseradish peroxidase were purchased from Pierce (Rockford, IL, USA).

## Authors' contributions

**MH **participated in the design of the study, carried out the artery preparations, organ culture, myograph experiments, analyzed the data and wrote most of the manuscript.

**PV **carried out the immunohistochemistry.

**ES **participated in the design of the study, participated in the myographs experiments and revised the manuscript.

**SB **carried out the Western blots and contributed to the writing and revision of the manuscript.

**LE **participated in the design of the study and revised the manuscript.

All authors read and approved the final manuscript.
